# Integration of legal aspects and human rights approach in palliative care delivery—the Nyeri Hospice model

**DOI:** 10.3332/ecancer.2016.656

**Published:** 2016-07-07

**Authors:** David Musyoki, Sarafina Gichohi, Johnson Ritho, Zipporah Ali, Asaph Kinyanjui, Esther Muinga

**Affiliations:** 1 Kenya Hospices and Palliative Care Association, PO Box 20854, 00202 Nairobi, Kenya; 2 Nyeri Hospice, PO Box 2246, 10140 Nyeri, Kenya

**Keywords:** legal aspects, human rights, palliative care, will

## Abstract

Palliative care is patient and family-centred care that optimises quality of life by anticipating, preventing, and treating suffering. Open Society Foundation public health program (2011) notes that people facing life-threatening illnesses are deeply vulnerable: often in severe physical pain, worried about death, incapacitation, or the fate of their loved ones.

Legal issues can increase stress for patients and families and make coping harder, impacting on the quality of care. In the absence of a clear legal provision expressly recognising palliative care in Kenya, providers may face numerous legal and ethical dilemmas that affect the availability, accessibility, and delivery of palliative care services and commodities. In order to ensure positive outcomes from patients, their families, and providers, palliative care services should be prioritised by all and includes advocating for the integration of legal support into those services. Palliative care service providers should be able to identify the various needs of patients and their families including specific issues requiring legal advice and interventions. Access to legal services remains a big challenge in Kenya, with limited availability of specialised legal services for health-related legal issues. An increased awareness of the benefits of legal services in palliative care will drive demand for easily accessible and more affordable direct legal services to address legal issues for a more holistic approach to quality palliative care.

## Introduction

The need for palliative care is increasing at a rapid pace due to the world’s ageing population and increases in cancer and other non-communicable diseases. According to World Health Organisation (WHO) Global Health estimates, there were approximately 54.6 million deaths worldwide in 2011. The great majority of deaths, 66%, are due to non-communicable diseases. There is an increased awareness of the need for palliative care for other chronic diseases or conditions such as HIV/AIDS, congestive heart failure, cerebrovascular disease, neurodegenerative disorders, chronic respiratory diseases, drug-resistant tuberculosis, and diseases of older people. However, there remains a huge unmet need for palliative care for these chronic life-limiting health problems in most parts of the world. Globally, in 2011, over 29 million people died from diseases requiring palliative care. The estimated number of people in the need of palliative care at the end of life is 20.4 million. The biggest proportion, 94%, corresponds to adults and 6% of all people in need of palliative care are children [1, 2].

Kenya has a current population of 43 million. Cancer ranks third as a cause of death after infectious and cardiovascular diseases. WHO estimates that there are about 82,000 new cancer cases in Kenya every year. According to the Kenyan National Cancer Control Strategy (NCCS) 2011–2016, cancer ranks third as a cause of death after infectious diseases and cardiovascular diseases. It is estimated that the annual incidence of cancer is about 28,000 cases and the annual mortality to be over 22,000. Kenya’s disease burden has continued to increase and up to 27% of hospice patients depend on palliative care. Kenya Hospices and Palliative Care Association (KEHPCA), the national organisation that coordinates palliative care in the country has continued to advocate for the integration of palliative care as one of the care options in the healthcare system [3].

According to WHO, palliative care is an approach that improves the quality of life of patients and their families facing the problem associated with life-threatening illness, through the prevention and relief of suffering by means of early identification and impeccable assessment and treatment of pain and other problems, physical, psychosocial, and spiritual.

The legal and human rights approach to palliative care in Kenya is a relatively new domain that needs to be expanded into all healthcare facilities, both private and public. This article looks at palliative care from a human rights perspective. Articles 1 and 2 of the Universal Declaration of Human Rights [5] state that all human beings are born free and equal in dignity and rights. The knowledge that palliative care is a human right deriving from the right to health has not been sufficiently appreciated in Kenya despite a robust constitutional framework guaranteeing the right to the highest attainable standard of health [8]. Additionally, legal support in palliative care is lacking in most parts of the country. Patients and their families rarely have access to legal practitioners who understand palliative care rights as these are few and far between. The legal fee charged by practitioners is also prohibitive.

Gwyther (2009) notes that advocates for both palliative care and human rights are increasingly recognising the link between these two powerful disciplines rooted in respect for human dignity [4].

Thus, there is a need to scale up access to legal support in palliative care to help patients and their families make plans for eventualities through will and powers of attorney. This need informed the partnership between the Kenya Hospices and Palliative Care Association (KEHPCA), its member hospices and palliative care providers, and Kenya Legal and Ethical Issues Network (KELIN), a human rights non-government organisation, to support the integration of legal aspects in palliative care through training community paralegals to offer legal support in palliative care on a voluntary basis, advocacy, and awareness creation on the issue. The community paralegals trained were drawn from various vocations: community health workers, nurses, clinicians, social workers, hospice administrators, lawyers, doctors, police officers, prison, and court paralegals.

KEHPCA is the national umbrella body for hospices and palliative care in Kenya. In line with its mission to promote and support acceptable, accessible and affordable quality palliative care for individuals and families by creating networks of informed and empowered institutions in Kenya, KEHPCA has embarked on a project to address legal issues in palliative care since 2010. The project observed that Nyeri Hospice initiates and fully integrates the legal aspects programme-targeting families and patients faced by life-threatening illnesses whom seek for services from the hospice.

Following the training, the paralegals in Nyeri prepared an action plan with deliverables and activities including the sensitisation of other staff members on legal aspects in palliative care, involvement of pro-bono lawyers in legal care, sensitisation of patients and families on legal issues and scheduling of a legal clinic every third Wednesday of the month. During the legal clinics, a pro bono advocate working closely with trained paralegals supports the clinic by providing legal counsel to the patients and family members on various issues including the process of writing valid will, custody of property and children, land and inheritance matter as per the Kenyan constitution, giving power of attorney among others. The staff members trained as paralegals support patients during the other days and refer the complicated cases to the lawyer and other legal institutions as appropriate.

Integration of the legal services can be replicated since the legal services are provided by a pro bono advocate as part of their expectation to give back to society. The paralegals trained are part of the enrolled staff members and therefore becomes part of their expected tasks. There is potential for other strategic partnerships with organisations that work on similar programmes.

With support from Open Society East Africa (OSIEA), KEHPCA received an approval from Salesforce foundation, which has a database, that enables the capturing of monthly data from the Nyeri Hospice. Plans are underway to analyse the data received to date. This process is being piloted with intermediate plans to bring on board three other hospices, namely Nairobi, Meru, and Coast. The success of this model will see further scale up of integrated legal work in all palliative care providers.

## Statistics

Sensitisation and awareness creation of legal issues in palliative care in Nyeri Hospice targeting patients and family members started on 16th February 2011 and has been going on to date. The hospice conducts a one -week professional training for healthcare workers every year for those in public and private healthcare facilities. During the training, a topic on legal aspects in palliative care is usually covered. Tables 1 and 2 provide the number of participants who have attended such fora.

Based on the activities outlined earlier, about 650 cases touching on legal issues have been dealt with successfully giving out results ranging from the following;

- Subdivision of property and transferring it to their heirs
- Handing over of bank accounts to spouses, children or relatives/friends when the patients are still alive
- Writing of valid will
- Granting of power of attorney (PoA) to spouses and family members
- Continuous dissemination of information to the patients, family members, relatives and the community.
- Intervention for the orphans and vulnerable children including seeking sponsor for their education programmes
- Trainings on legal issues for healthcare professionals
- Reduction in succession and inheritance conflicts in families and in communities.

## Challenges of the programme

Challenges that befall the legal aspects programme in palliative care include a high turnover of trained lawyers, few numbers of para-legal staff members, an increasing burden of tasks that require their expertise, limited resource materials on legal issues to train the patients with, especially on will writing, power of attorney, patients’ rights, succession and inheritance, and limited funding to enhance the programme. Other challenges are the lack of motivation of lawyers willing to volunteer to participate in day care sessions and community education sessions and cultural inhibitions and misconceptions on will-writing, succession, and inheritance. All the above pose a challenge to the sustainability of such a noble aspect of care

## Conclusion

There are many palliative care developments taking place in Kenya including advocacy and integration of palliative care in healthcare services. Strategic planning in the various projects is important to enhance the sustainability of such programmes. Callaway *et al*. (2007) note that financial problems are common to most of the palliative care programmes within developing countries like Kenya. [1] She further recommends both national and international foundations and agencies that provide funding for hospice and palliative care activities in the developing world need to identify those countries with the potential capacity and political will to initiate palliative care services. This will ensure a strong financial base to make such programmes successful, hence improving the qualities of life of the patients and families reached.

One of the recipients of care at the Nyeri Hospice said the following, *‘I have learned that I am not a victim at the legal clinic, I have learned how to make a will, and that a will is not just for the terminally ill- it’s everyone’s right. Now that I’ve inherited my husband’s property, I understand that the title deeds need to be put in my name, if I want to leave my land to my grandchildren’.*

## Funding

The writing of this paper received no specific grant from any funding agency in the public, commercial, or not-for-profit sectors.

## Figures and Tables

**Table 1.** Number of persons who participated in various legal awareness sessions at Nyeri Hospice.

Year	Patients	Relatives	Community members	Total
2011	74	166	966	1206
2012	108	114	1195	1417
2013	77	136	1380	1593
2014	48	85	2443	2576
2015	104	139	3657	3900
**TOTALS**	**411**	**640**	**9641**	**10,692**

**Table 2.** Health care professionals training.

Year	Number of trainees
2011	18
2012	25
2013	16
2014	24
2015	10
**Total**	**93**

Number of trainees sensitised on legal aspects
